# Effect of Cellulose Solvents on the Characteristics of Cellulose/Fe_2_O_3_ Hydrogel Microspheres as Enzyme Supports

**DOI:** 10.3390/polym12091869

**Published:** 2020-08-19

**Authors:** Saerom Park, Yujin Oh, Dahun Jung, Sang Hyun Lee

**Affiliations:** Department of Biological Engineering, Konkuk University, Seoul 05029, Korea; angel4y@naver.com (S.P.); so01068@naver.com (Y.O.); 1wjdekgns@naver.com (D.J.)

**Keywords:** cellulose, solvent, hydrogel, microsphere, enzyme, immobilization

## Abstract

Cellulose hydrogels are considered useful biocompatible and biodegradable materials. However, as few cellulose-dissolving solvents can be used to prepare cellulose hydrogel microspheres, the use of unmodified cellulose-based hydrogel microspheres for enzyme immobilization remains limited. Here, we prepared cellulose/Fe_2_O_3_ hydrogel microspheres as enzyme supports through sol-gel transition using a solvent-in-oil emulsion. Cellulose-dissolving solvents including 1-ethyl-3-methylimidazolium ([Emim][Ac]), an aqueous mixture of NaOH and thiourea, tetrabutylammonium hydroxide, and tetrabutylphosphonium hydroxide were used to prepare regular shaped cellulose/Fe_2_O_3_ microspheres. The solvent affected microsphere characteristics like crystallinity, hydrophobicity, surface morphology, size distribution, and swelling properties. The immobilization efficiency of the microspheres for lipase was also significantly influenced by the type of cellulose solvent used. In particular, the lipase immobilized on cellulose/Fe_2_O_3_ microspheres prepared using [Emim][Ac] showed the highest protein loading, and its specific activity was 3.1-fold higher than that of free lipase. The immobilized lipase could be simply recovered by a magnet and continuously reused.

## 1. Introduction

Hydrogels are polymeric materials with three-dimensional network structures formed by hydrophilic polymers, which are swollen with a large volume of water [1,2]. Recently, because of their inherent biocompatibility, biodegradability, and non-toxicity, naturally derived biopolymer-based hydrogels have attracted tremendous attention in the biotechnological field for fabrication of protein supports, drug delivery carriers, tissue engineering scaffolds, biosensors, wound healing materials, etc., as well as for environmental applications such as fabrication of adsorbents for toxic compounds and supports for cell immobilization [3,4]. Particularly, hydrogels of polysaccharides such as chitosan, alginate, starch, carrageenan, agarose, cellulose derivatives, and proteins such as collagen and gelatin have been employed as enzyme supports. Various enzymes such as oxidoreductases, hydrolases, lyases, and isomerases have been successfully immobilized on various biopolymer-based hydrogels [5,6,7,8,9].

Cellulose, a major component of plants that is also present in bacteria, fungi, algae, and some aquatic animals, is the most abundant biopolymer on earth. It is a linear polysaccharide formed by d-glucose molecules bound through *β*-(1-4)-glycosidic [10,11]. Cellulose hydrogels are considered as useful biocompatible and biodegradable materials for application in the medical, environmental, agricultural, and textile fields because of their excellent mechanical, chemical, and thermal resistance [1]. Cellulose hydrogels can be prepared by the dissolution of cellulose in cellulose-dissolving solvents, followed by regeneration with anti-solvents [12]. The abundant hydroxyl groups of cellulose can form hydrogen bonding networks during the regeneration process. However, the development of unmodified cellulose-based hydrogels has been limited because of the difficulty in dissolving cellulose using general solvents. 

Various cellulose-dissolving solvents have been developed, such as *N*-ethylpyridinium chloride, LiCl/*N*,*N*-dimethylacetamide solution, *N*-methylmorpholine *N*-oxide monohydrate, and ammonium fluoride/dimethyl sulfoxide. However, these solvents have problems such as toxicity, high energy consumption during the dissolution process, and high cost [13,14,15]. Recently, novel cellulose-dissolving green solvents such as ionic liquids, alkylammonium hydroxide, and alkylphosphonium hydroxide were developed to overcome the disadvantages of previous cellulose solvents [16,17,18]. The development of various cellulose-dissolving solvents has triggered research on the application of unmodified cellulose-based hydrogels. 

Micrometer-sized cellulose hydrogel beads have potentials for the development of adsorbents, drug delivery carriers, chromatographic resins, and enzyme supports because of their high surface area and hydrophilic properties [19,20,21]. Cellulose hydrogel microspheres are conventionally prepared through a sol-gel transition method using a solvent-in-oil emulsion. Previous studies have shown that the size and uniformity of the prepared cellulose hydrogel microspheres were affected by the ratio of solvent to oil, surfactant concentration, cellulose concentration, and stirring speed [2,22,23]. Peng et al. prepared cellulose/chitosan/Fe_3_O_4_ hydrogel microspheres for Cu(II) adsorption using an emulsion of 1-butyl-3-methylimidazolium chloride and vacuum oil [24]. Li et al. prepared cellulose/guar gum/Fe_3_O_4_ hydrogel microspheres as a drug carrier [25]. Meng et al. prepared cellulose/lignin/Fe_3_O_4_ hydrogel microspheres for the adsorption of dyes and heavy metals [26]. 

Recently, we prepared cellulose hydrogel microspheres using 1-ethyl-3-methylimidazolium acetate ([Emim][Ac]) as enzyme supports [2]. The lipase immobilized on cellulose hydrogel microspheres showed a much higher loading efficiency, immobilization yield, and specificity constant than lipase immobilized on microcrystalline cellulose or millimeter-sized hydrogel beads, because of higher surface area and favorable interactions of cellulose microspheres. Furthermore, we prepared cellulose/biopolymer/Fe_3_O_4_ hydrogel microspheres to modify the adsorption capacity for enzymes [12]. Liu et al. prepared cellulose/chitosan/Fe_3_O_4_ hydrogel microspheres for the immobilization of glucose oxidase [27]. Xue et al. studied the immobilization of lysozyme on cellulose/Fe_3_O_4_ microspheres [28]. Zhang et al. prepared polyporous magnetic cellulose to immobilize lipase [29]. Thus, cellulose hydrogel microspheres have the potential to be applied as enzyme supports. However, the use of unmodified cellulose-based hydrogel microspheres for enzyme immobilization remains limited owing to the difficulty of preparation and because few cellulose-dissolving solvents could be used to prepare cellulose hydrogel microspheres.

In this study, cellulose/Fe_2_O_3_ hydrogel microspheres were prepared in an environmentally friendly way through the sol-gel transition process using a solvent-in-oil emulsion. Various green solvents including [Emim][Ac], an aqueous mixture of NaOH/thiourea (9.3%/7.3%), 40% tetrabutylammonium hydroxide, and 40% tetrabutylphosphonium hydroxide were used to dissolve cellulose and form an emulsion with non-toxic soybean oil. The effect of cellulose solvent on the characteristics of the prepared cellulose hydrogel microspheres was investigated by analyzing the crystallinity, surface morphology, size distribution, and swelling properties of cellulose microspheres. In particular, the effect of the cellulose solvent on the immobilization efficiency of cellulose/Fe_2_O_3_ hydrogel microspheres as enzyme supports was investigated by measuring the protein loading, specific activity, and reusability of immobilized lipases.

## 2. Materials and Methods

### 2.1. Materials

Microcrystalline cellulose (MCC), 1-ethyl-3-methylimidazolium acetate ([Emim][Ac]), 40% tetrabutylammonium hydroxide (TBAH), 40% tetrabutylphosphonium hydroxide (TBPH), thiourea, Span^®^ 85, iron oxide (Fe_2_O_3_), lipase from *Candida rugosa* (Type VII, 1176 U/mg), *p*-nitrophenyl butylate, and *p*-nitrophenol were purchased from Sigma Aldrich (St. Louis, MO, USA). Sodium hydroxide, ethanol, hexane, isopropyl alcohol, Congo red, and water (HPLC grade) were obtained from Samchun Pure Chemical (Gyeonggi-do, Korea). Soybean oil was purchase from SAJO Daerim (Incheon, Korea). All other chemicals used were of analytical grade and were used without further purification.

### 2.2. Preparation of Regenerated Cellulose Films and Magnetic Cellulose Microspheres

To develop regenerated cellulose films, [Emim][Ac], NaOH/thiourea (9.3%/7.4%) aqueous solution (NaTU), 40% TBAH, and 40% TBPH were used as cellulose-dissolving solvents. MCC (10%, *w/v*) was dissolved in each cellulose solvent, and the transparent cellulose solution was subsequently cast on glass slides using a micrometer film applicator/1117 (Mitutoyo Corp., Kawasaki, Japan); then, cellulose was regenerated with anti-solvent (Table 1).

The regenerated cellulose film prepared using [Emim][Ac] (Cell-IL) was washed with water until the optical density of the washing solution was not detected at 211 nm. Other regenerated cellulose films were neutralized and washed with water. The fabricated cellulose hydrogel films were dried at room temperature. 

Magnetic cellulose microspheres were prepared by sol-gel transition using a solvent-in-oil emulsion. First, 0.25% (*w/v*) Fe_2_O_3_ was dispersed in each cellulose solvent (3 mL) by sonication. Then, MCC (4%, *w/v*) was dissolved in the Fe_2_O_3_/solvent mixture under the dissolution conditions shown in Table 1. The cellulose solution containing Fe_2_O_3_ was emulsified with soybean oil (7.5 mL) under mechanical stirring for 2 h. For emulsification of the NaTU mixture with oil, 5% Span^®^ 85 was added for efficient emulsification. Next, an anti-solvent was added to the emulsified solvent/oil solution under stirring to regenerate cellulose, and then, the mixture was stirred for an additional 1 h to obtain magnetic cellulose microspheres. When magnetic cellulose microspheres were prepared using [Emim][Ac], ethanol instead of water was used as an anti-solvent because of the difficulty of phase separation of oil and [Emim][Ac]/water. After the regeneration process, the prepared magnetic cellulose microspheres were harvested using a neodymium magnet, and the liquid phase was removed. The collected microspheres were washed with water by the above described washing process of cellulose films. Finally, the prepared magnetic cellulose microspheres were stored in HPLC-grade water until further use.

### 2.3. Characterization of Regenerated Cellulose Films and Magnetic Cellulose Microspheres

Changes in functional groups in the regenerated cellulose films prepared using different types of cellulose solvents were investigated by Fourier-transform infrared spectroscopy (FT-IR). The dried cellulose films were ground using a homogenizer. The FT-IR spectra of the cellulose films were determined by an average of 16 scans per spectrum in the range 4000–600 cm^−1^ (resolution of 4 cm^−1^ and scanning interval of 1 cm^−1^) using an FT/IR-4100 type A spectrometer (Jasco International Co. Ltd., Tokyo, Japan).

X-ray diffraction (XRD) patterns of regenerated cellulose films were analyzed using an X-ray diffractometer (D8 Advance, Bruker, Germany). The samples were scanned in a 2 theta(θ) range 2–40° at 40 kV and 40 mA. The crystallinity index (CrI) of cellulose was calculated using the height difference method [30,31]. The d-spacings were calculated using Bragg’s law. The crystallite sizes of cellulose were determined using Scherrer’s law [32].

To verify the formation of the magnetic cellulose microspheres, the microspheres were stained as hydrogel forms with Congo red and were observed by optical microscopy. Furthermore, the magnetic cellulose microspheres were frozen overnight at −70 °C and dried at −80 °C for 12 h under vacuum. The freeze-dried microspheres were sputter-coated with gold for surface observation using a scanning electron microscope (SEM; SUPRA 55VP, Carl Zeiss, Jena, Germany). The size distribution of magnetic cellulose microspheres in the hydrogel and freeze-dried forms was detected using a laser diffraction particle size analyzer (DLS; Mastersizer 2000, Malvern PANalytical, Malvern, UK). The equilibrium swelling degree (ESD) of magnetic cellulose microspheres was determined after swelling microspheres (25 mg dry weight) in distilled water for 24-h incubation. The ESD (mL/g) was defined as the ratio of the swollen volume to the mass of the dried microsphere.

### 2.4. Immobilization of Lipase on Magnetic Cellulose Microspheres

Ten milligrams of lipase powder were dissolved in 1 mL of 0.1 M sodium phosphate buffer (pH 7), and then, the supernatant containing 1.2 mg/mL protein after centrifugation was used for immobilization. To adsorb the lipase on the magnetic cellulose microspheres, 5 mg (as dry weight) cellulose/Fe_2_O_3_ hydrogel microspheres were added into the 40 μg/mL lipase solution, and the adsorption process was performed in a water bath at 25 °C with shaking at 120 rpm for 1 h. The magnetic cellulose microspheres containing lipase were recovered using a magnet block, and then, the microspheres were washed with 0.1 M sodium phosphate buffer (pH 7) to remove weakly adsorbed proteins. Finally, the immobilized lipases were stored in 0.1 M sodium phosphate buffer (pH 7) until further use. The quantity of adsorbed protein was determined by measuring the difference between the initial protein content in the buffer before immobilization and the remaining protein content in the buffer after immobilization. The protein concentrations were analyzed using the Micro BCA^TM^ protein assay kit. 

### 2.5. Determination of Lipase Activity

To analyze the hydrolytic activity of the immobilized lipase, spectrophotometry using *p*-nitrophenyl butyrate as a substrate was performed. The lipase immobilized on cellulose/Fe_2_O_3_ hydrogel microspheres (0.1 mg of dry weight) was dispersed in 9.5 mL of 0.1 M sodium phosphate buffer (pH 7). The reaction was started by adding 0.5 mL of 10 mM *p*-nitrophenyl butyrate in isopropanol to the enzyme mixture [33,34]. The reaction was carried out in a water bath at 25 °C with a shaking speed of 120 rpm. To determine the initial rate of the hydrolytic reaction catalyzed by the immobilized lipase, aliquots of 0.3 mL were periodically withdrawn and diluted with 0.3 mL acetonitrile. The samples were centrifuged; then, the content of *p*-nitrophenol, which was formed as a product in the supernatant, was determined by measuring the absorbance at 400 nm. The initial rates of the immobilized lipase were measured in triplicate. 

### 2.6. Reuse of Immobilized Lipase

The reusability of the lipase immobilized on the magnetic cellulose microspheres was measured through repeated use in the hydrolysis reaction of *p*-nitrophenyl butyrate. The immobilized lipase after hydrolysis reaction was recovered using a magnet and then washed with 0.1 M sodium phosphate buffer (pH 7). The residual activity of the immobilized lipase was measured when the magnetic cellulose microspheres were reused ten times. 

## 3. Results and Discussion

### 3.1. Effect of Cellulose Solvent on the Characteristics of Regenerated Celluloses

To investigate the effect of the cellulose-dissolving solvents on the characteristics of regenerated cellulose, 10% MCC was dissolved in [Emim][Ac], as a model ionic liquid, and alkali solutions including NaTU, TBAH, and TBPH. When MCC was dissolved in cellulose solvents, all cellulose solutions became transparent and viscous; moreover, no cellulose crystals were observed under a polarization microscope (BX-41, Olympus, Japan). 

**Figure 1:**
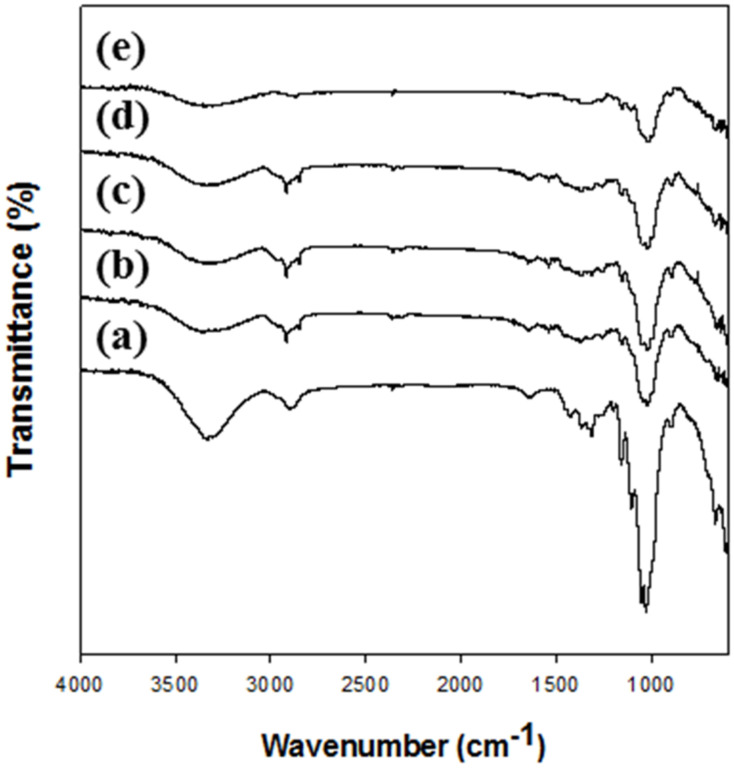
FT-IR spectra of Microcrystalline cellulose (MCC) and regenerated celluloses. (**a**) MCC, (**b**) Cell-IL, (**c**) Cell-NaTU, (**d**) Cell-TBAH, and (**e**) Cell-TBPH.

The changes in the functional groups of cellulose after the dissolution and regeneration were investigated using FT-IR (Figure 1). The FT-IR spectra (Figure 1a) of native cellulose (MCC) showed a broad peak attributed to O–H stretching vibration of hydrogen bonding at 3000–3600 cm^−1^, a CH_2_ group peak at approximately 2900 cm^−1^, a peak for OH bending of water in cellulose at approximately 1640 cm^−1^, a peak of intermolecular hydrogen bonding of the C4 group at approximately 1430 cm^−1^, a peak of CH deformation vibration at approximately 1370 cm^−1^, and a peak of C–O–C asymmetric stretching at approximately 1160 and 900 cm^−1^, indicating characteristics of cellulose [31,35,36,37]. In the spectrum of regenerated celluloses, the same peaks denoting characteristics of cellulose were also observed, but some peaks had shifted. Especially, the peaks in the range 3000–3600 cm^−1^ were shifted because of the breaking of hydrogen bonds between hydroxyl groups; in addition, peak shifts at approximately 1160 and 900 cm^−1^ and deficit of peak at approximately 1430, 1370–1315, 1105, and 1053 cm^−1^ were observed. These changes indicate that the cellulose I structure was changed to that of cellulose II [31,38,39,40,41]. Moreover, a small peak occurred at approximately 990 cm^−1^ in the regenerated cellulose spectrum, indicating C–O stretching vibration in the amorphous region [41]. These results show that there were no chemical changes such as the introduction and deficit of functional groups after the regeneration of cellulose [42]. However, the cellulose I structure of MCC was changed to a cellulose II structure after the regeneration. 

**Figure 2:**
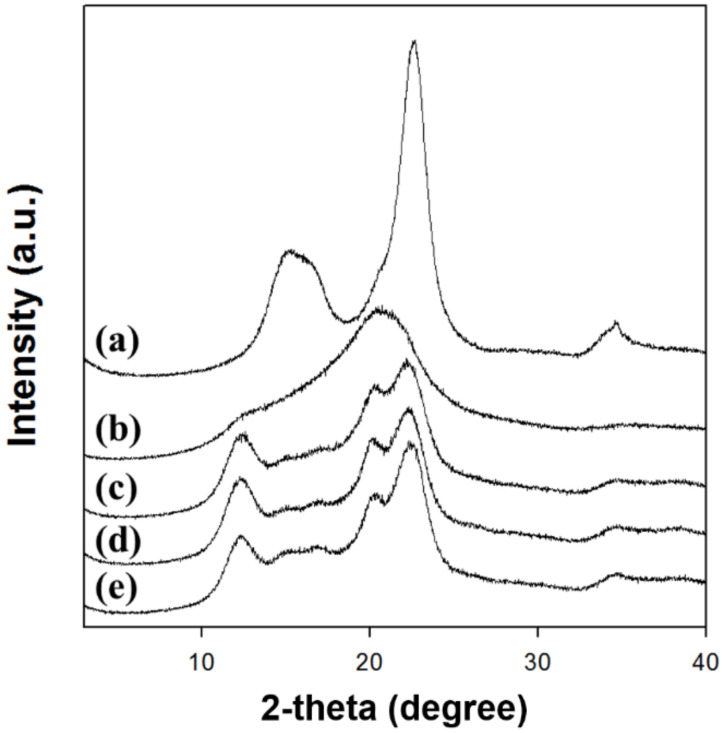
XRD patterns of MCC and regenerated celluloses. (**a**) MCC, (**b**) Cell-IL, (**c**) Cell-NaTU, (**d**) Cell-TBAH, and (**e**) Cell-TBPH.

The XRD analysis clearly depicted that the type of solvent for dissolving cellulose highly affected the crystalline structure of regenerated cellulose (Figure 2). The XRD patterns (Figure 2a) of native cellulose showed diffraction peaks at 2θ = 15.3°, 16.1°, and 22.7°, indicating the cellulose I crystal structure [32,39]. The XRD patterns of cellulose regenerated using [Emim][Ac] showed that the main diffraction peak was shifted and widened at 2θ = 20.7° (Figure 2b). The existence of peaks at approximately 2θ = 20° suggested the conversion of crystal structure into amorphous state [41,43,44]. In contrast, the cellulose regenerated using alkali solutions including NaTU, TBAH, and TBPH displayed diffraction peaks at 2θ = 12.3°, 14–18°, 20.4°, and 22.3° corresponding to the cellulose II crystal structure [45]. Therefore, the regenerated cellulose formed amorphous or cellulose II crystal structures depending on the kind of cellulose-dissolving solvent.

The CrI values of native cellulose and regenerated celluloses were calculated using the peak height method (Table 2). The CrI values of the regenerated celluloses were lower than those of the native cellulose. The CrI value of cellulose regenerated using [Emim][Ac] could not be determined by the peak height method because of its amorphous structure. The decrease in CrI value was caused by the rearrangement of hydrogen bonds which occurred during the dissolution and reconstruction of cellulose. The d-spacing and crystallite size were also calculated for the [200] planes of celluloses (Table 2). Furthermore, the d-spacing increased from 0.392 to 0.426 nm and the crystallite size decreased from 4.2 to 1.2 nm after cellulose regeneration. The d-spacing (inter-planar spacing) is related to the hydrophobic interaction of cellulose. Therefore, the increase in d-spacing of regenerated cellulose revealed that the crystal structure was weaker than that of native cellulose because of the decrease in hydrophobic bonds of cellulose during the regeneration process [46,47]. In a previous report, the alkali solution treatment of cellulose with high crystallinity (CrI > 0.67) reduced the crystallinity and crystallite size [46]. Kim et al. reported that the crystallinity increased with increasing crystallite size [48].

The crystallinity and crystallite sizes of the regenerated cellulose increased and its d-spacing decreased in the following order: Cell-IL < Cell-NaTU < Cell-TBAH < Cell-TBPH. These results indicate that the crystalline structure of regenerated cellulose highly depends on the type of cellulose-dissolving solvent. Alves et al. also reported that the use of alkaline solutions to dissolve and regenerate cellulose can decrease the crystallinity and change the structure of cellulose from cellulose I to cellulose II and an amorphous structure [49]. 

### 3.2. Effect of Cellulose Solvent on the Characteristics of Magnetic Cellulose Microspheres

**Figure 3:**
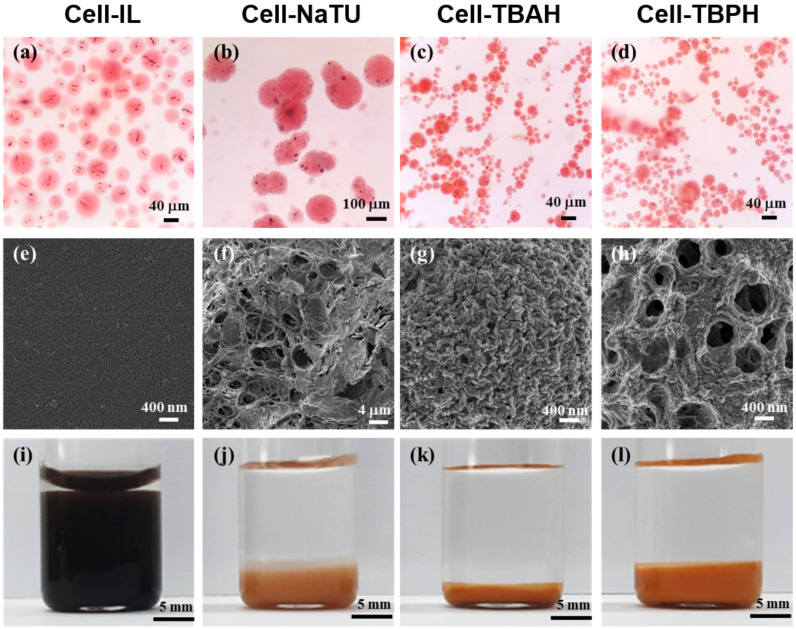
Optical microscopic images of magnetic cellulose microspheres stained with Congo red (**a**–**d**); SEM images of freeze-dried microspheres (**e**–**h**); and photographic images of microspheres (dry weight, 25 mg after freeze-drying) dispersed in distilled water after 24-h incubation (**i**–**l**). (**a**,**e**,**i**): Cell-IL, (**b**,**f**,**j**): Cell-NaTU, (**c**,**g**,**k**): Cell-TBAH, and (**d**,**h**,**l**): Cell-TBPH.

The magnetic cellulose microspheres were fabricated by sol-gel transition using and oil-based emulsion. All microspheres were prepared as spherical shapes (Figure 3). Cell-IL, Cell-TBAH, and Cell-TBPH microspheres obtained without the addition of surfactant were spherical in shape; however, NaTU microspheres were only obtained when 5% of surfactant was added.

The surface morphology of the freeze-dried magnetic cellulose microspheres was highly dependent on the type of cellulose solvent. In the freeze-drying process, the Cell-IL microspheres retained their spherical shapes until freezing; however, the spherical shape of Cell-IL microspheres was destroyed during the drying process. In contrast, the Cell-NaTU, Cell-TBAH, and Cell-TBPH microspheres preserved their spherical shapes during freeze-drying. The surface of Cell-IL microspheres was smooth, while the microspheres prepared using alkali solvents showed porous surface structures. The Cell-NaTU and Cell-TBPH microspheres had a reticulated porous structure and an open pore structure, respectively. The surface of Cell-TBAH microspheres was rugged and had fine and dense pores. The pore size of the Cell-NaTU microspheres was found to be approximately 0.1–1% of the size of microspheres. The pore diameter of Cell-TBAH was less than 100 nm, corresponding to approximately 1% of the diameter of microspheres. The pore diameter of the Cell-TBPH microspheres was approximately 400 nm, which was approximately 5% of the diameter of microspheres. 

**Figure 4:**
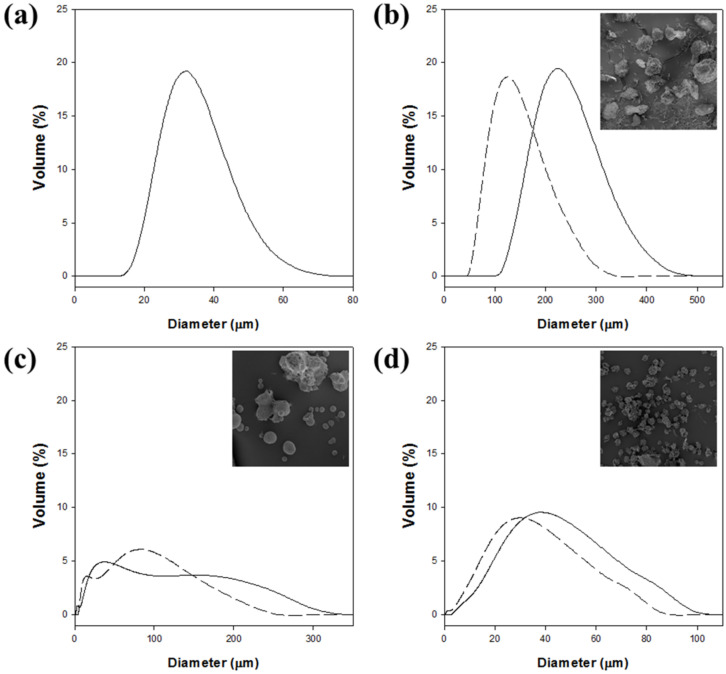
Size distribution of the magnetic cellulose microbeads before (solid line) and after (dashed line) freeze-drying. (**a**) Cell-IL, (**b**) Cell-NaTU, (**c**) Cell-TBAH, and (**d**) Cell-TBPH.

The size distribution of magnetic cellulose microspheres was also highly affected by the type of cellulose solvent (Figure 4 and Table 3). The mean diameter of magnetic cellulose microspheres in the hydrogel form ranged from 33 to 232 μm. The sizes of magnetic microspheres increased in the following order: Cell-IL < Cell-TBPH < Cell-TBAH < Cell-NaTU. The Cell-IL microspheres showed the smallest mean diameter and the narrowest size distribution. The mean diameter of Cell-TBPH microspheres was similar to that of the Cell-IL microspheres, but the size distribution of Cell-TBPH was wider than that of Cell-IL. The average diameters of Cell-TBAH and Cell-NaTU microspheres were 2.1- and 7.0-fold higher than those of Cell-IL microspheres, respectively. 

The size distribution of magnetic cellulose microspheres after freeze-drying was also measured to understand the swelling property of cellulose microspheres (Figure 4 and Table 3). The average diameters of microspheres were highly decreased after freeze-drying. Different swelling ability of prepared cellulose microsphere could be caused by surface porosity and water accessibility. The size distribution of the Cell-IL microspheres could not be measured because the microsphere structure was destructed. The mean diameters of the freeze-dried Cell-NaTU, Cell-TBAH, and Cell-TBPH microspheres were reduced by 42%, 12%, and 24%, compared with those of hydrogel microspheres, respectively. The higher swelling property of Cell-NaTU microspheres could be understood by the lower crystallinity of Cell-NaTU, compared with those of Cell-TBAH and Cell-TBPH.

The equilibrium swelling degree (ESD) of magnetic cellulose microspheres was determined to understand the wettability and hydrophilicity of microspheres (Figure 3 and Table 3). The Cell-IL microspheres showed the highest ESD, and the value was 6 times higher than that of Cell-TBAH microspheres which showed the lowest ESD. The high swelling degree of the Cell-IL microsphere can be understood by its low crystallinity and amorphous structure. The weak internal hydrogen bonding of Cell-IL microspheres can enhance the external hydrogen bonding between hydroxyl groups of cellulose and water molecules. The lowest ESD of Cell-TBAH microspheres among cellulose microspheres prepared using alkali solution can be attributed to its dense surface morphology due to the smallest average pore size of less than 100 nm. The Cell-TBAH microspheres also showed the lowest diameter reduction after freeze-drying (Table 3). The ESD of Cell-NaTU microspheres was similar to that of Cell-TBPH microspheres, although the size reduction (42%) after freeze-drying of Cell-NaTU was much higher than that (24%) of Cell-TBPH and the mean diameter of freeze-dried Cell-NaTU was 4.9 times higher than that of Cell-TBPH. 

These results indicate that the crystallinity, hydrophobicity/hydrophilicity, surface morphology, and swelling property of the magnetic cellulose microspheres are highly affected by the solvent used to prepare the cellulose microspheres.

### 3.3. Immobilization of Lipase on Magnetic Cellulose Microspheres

To investigate the possibility of applying the magnetic cellulose microspheres as enzyme supports, lipase from *Candida rugosa* was physically adsorbed on the cellulose microspheres, and the properties of the immobilized lipase were analyzed (Table 4). 

The amount of protein adsorbed on magnetic cellulose microspheres was mainly affected by the average diameter of microspheres, and the adsorbed content of protein increased as the size of cellulose microspheres decreased. The highest content of protein was loaded on Cell-IL microspheres which had the smallest mean diameter, whereas the content of protein loaded on Cell-NaTU microspheres, which had the largest mean diameter, was 56% of that loaded on Cell-IL microspheres. 

The activity of lipase immobilized on magnetic cellulose microspheres was also increased as the size of microspheres decreased. Higher protein loading induced higher activity of immobilized lipase. Interestingly, the lipase immobilized on Cell-IL microspheres showed significantly higher activity than the lipases immobilized on the cellulose microspheres prepared using alkali solutions. The activity of lipase immobilized on Cell-IL microspheres was 12-fold higher than that immobilized on Cell-NaTU microspheres. Exceptionally high activity of lipase immobilized on Cell-IL microspheres could have been caused by the high protein loading and favorable interactions between lipase and the microsphere surfaces.

The specific activity and relative activity of the lipase immobilized on magnetic cellulose microspheres were determined to understand the recovered activity of lipase after immobilization (Table 4). The specific activity was defined as the ratio of the activity to protein content. The relative activity (%) was defined as the ratio of the specific activity of immobilized lipase to the specific activity of free lipase. When the lipase immobilized on magnetic cellulose microspheres prepared using alkali solutions (NaTU, TBAH, and TBPH) were compared, the lipase immobilized on Cell-TBPH showed the highest relative activity, indicating that the characteristics such as small diameter and large ESD of Cell-TBPH microspheres are more favorable to retain the activity of lipase. In general, the relative activity of immobilized enzyme was less than 100% because of the steric hinderance of immobilized enzyme and limited mass transfer of substrate into the active site of immobilized enzyme. However, interestingly, the relative activity of the lipase immobilized on Cell-IL microspheres was 3.1-fold higher than that of free lipase. The significantly enhanced specific activity of lipase immobilized on Cell-IL microspheres can be understood by the conformational change of the lid-opening and the favorable interactions between lipase and the microsphere surfaces. The lid structure of *Candida rugosa* lipase should be open to activate the active site of lipase, and lid-opening could have occurred by the hydrophobic interaction with Cell-IL microspheres. These results show that magnetic cellulose microspheres have potential to be applied as enzyme supports. In particular, the solvent used to prepare magnetic cellulose microspheres can highly affect the physical characteristics such as size, surface morphology, swelling degree, and the immobilization efficiency of microspheres.

**Figure 5:**
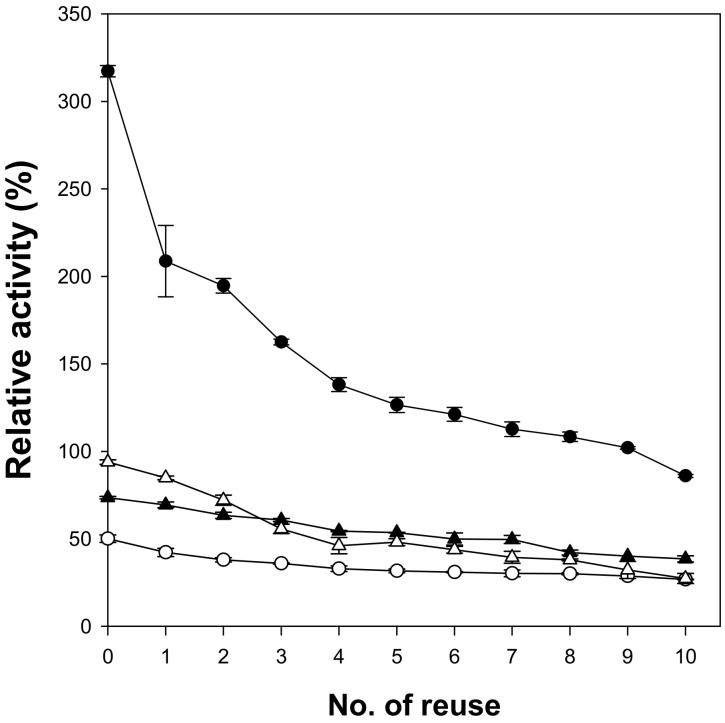
Relative activity of lipases immobilized on the magnetic cellulose microspheres after reuse. (●) Cell-IL, (○) Cell-NaTU, (▲) Cell-TBAH, and (△) Cell-TBPH.

When microspheres are used as enzyme supports, easy separation of enzymes after the reaction is an important issue. Magnetic cellulose microspheres prepared in this work were easily recovered using a magnet in 10 min and were successfully reused. The reusability of lipase immobilized on magnetic cellulose microspheres was analyzed by measuring the residual activity after 10-time reuse (Figure 5). To investigate the usefulness of the magnetic cellulose microspheres as enzyme supports, immobilized lipases with similar content of protein were used and their relative activities were compared with those of free lipase (100%). The lipase immobilized on Cell-IL microspheres showed the highest initial activity because of the highest specific activity. After the first cycle, the lipase immobilized on Cell-IL showed the highest activity loss. It may be caused by the release of weakly adsorbed lipase. The first adsorption step of lipase on cellulose microspheres is the surface interaction and the next step is the diffusion of lipase into the inside of hydrogel. Most lipase immobilized on Cell-IL may exist on the surface of cellulose microspheres due to low accessibility of lipase into the hydrophilic inside of Cell-IL. Another possible reason is the loss of microspheres during the recovering process due to the smallest size of Cell-IL. The residual activity of lipase immobilized on Cell-IL microspheres became approximately 30% of initial activity after 10-time reuse; however, the immobilized lipase still showed the highest relative activity of 85% among the tested magnetic cellulose microspheres. The lipase immobilized on Cell-NaTU and Cell-TBAH microspheres retained more than 50% of its initial activity after 10-time reuse. These results show that the type of cellulose solvent used to prepare magnetic cellulose microspheres can also affect the stability of immobilized lipase.

## 4. Conclusions

In this study, to use cellulose/Fe_2_O_3_ hydrogel microspheres as enzyme supports, the microspheres were successfully prepared using various cellulose-dissolving green solvents including [Emim][Ac], NaTU, TBAH, and TBPH. The cellulose/Fe_2_O_3_ hydrogel microspheres of regular sphere shape could be environmental-friendly prepared from the soybean oil-based emulsion without the help of a surfactant. The characteristics of the prepared cellulose microspheres were highly dependent on the type of solvent used to dissolve the cellulose. The crystallinity of the regenerated cellulose was lower than that of pristine cellulose; particularly, the structure of cellulose regenerated from [Emim][Ac] was completely changed to an amorphous structure. Physical properties such as mean diameter, surface morphology, swelling degree, and hydrophilicity/hydrophobicity of the magnetic cellulose microspheres were changed using different cellulose solvents. The different characteristics of magnetic cellulose microspheres significantly affected the immobilization efficiency of lipase from *C. rugosa*. The loaded protein content and the activity of immobilized lipase increased as the size of microspheres decreased. Especially, magnetic cellulose microspheres prepared using [Emim][Ac] could efficiently immobilize the lipase with the highest protein loading among the tested microspheres and with higher specific activity than free lipase. The magnetic cellulose microspheres prepared using NaTU and TBAH showed high lipase stability. These results indicate the efficacy of using magnetic cellulose microspheres as enzyme supports and the importance of cellulose solvents to prepare microspheres. Various characteristics of cellulose hydrogel microspheres can be controlled by changing the cellulose solvent to develop efficient enzyme supports.

## Figures and Tables

**Table 1:** Dissolution and regeneration conditions to prepare regenerated cellulose hydrogels.

Regenerated Cellulose	Dissolution Conditions			Anti-Solvent
	Solvent	Temperature (°C)	Time (min)	
Cell-IL	[Emim][Ac]	100	120	Ethanol
Cell-NaTU	NaTU	8	6	DW
Cell-TBAH	TBAH	25	120	DW
Cell-TBPH	TBPH	25	120	DW

**Table 2:** XRD parameters of MCC and regenerated celluloses.

Regenerated Cellulose	CrI	D-Spacing (200) of the Planes (nm)	Crystallite Sizes (nm)
MCC	0.806	0.392	4.2
Cell-IL	ND	0.426	1.2
Cell-NaTU	0.549	0.4	1.7
Cell-TBAH	0.572	0.398	1.9
Cell-TBPH	0.618	0.396	2

**Table 3:** Mean diameters and swelling degrees of magnetic cellulose microspheres.

Magnetic Cellulose Microspheres	Mean Diameter of Hydrogel Beads (μm)	Mean Diameter of Freeze-Dried Beads (μm)	Equilibrium Swelling Degree (mL/g)
Cell-IL	33.0 ± 0.4	ND	99.3
Cell-NaTU	231.8 ± 3.3	133.9 ± 0.5	31.4
Cell-TBAH	69.4 ± 1.2	61.3 ± 0.1	16.3
Cell-TBPH	36.3 ± 0.0	27.6 ± 0.2	35.2

**Table 4:** Activity of lipases immobilized on the magnetic cellulose microspheres.

Magnetic Microspheres	Loaded Protein (μg/mg Beads)	Activity (×10^−3^ μmol/min)	Specific Activity (μmol/min/mg Protein)	Relative Activity * (%)
Cell-IL	8.9 ± 0.5	121.9 ± 10.8	125.2 ± 12.5	314.4
Cell-NaTU	5.0 ± 0.6	10.0 ± 0.2	17.1 ± 2.1	43.1
Cell-TBAH	6.6 ± 0.4	21.0 ± 0.6	30.4 ± 3.3	76.4
Cell-TBPH	7.7 ± 0.2	28.1 ± 1.1	33.9 ± 2.5	85.0
Free lipase			39.8 ± 1.7	100.0

* Relative activity = (specific activity of immobilized lipase/specific activity of free lipase) × 100.
